# Large-scale effects of migration and conflict in pre-agricultural groups: Insights from a dynamic model

**DOI:** 10.1371/journal.pone.0172262

**Published:** 2017-03-08

**Authors:** Francesco Gargano, Lucia Tamburino, Fabio Bagarello, Giangiacomo Bravo

**Affiliations:** 1 Department of Energy, Engineering of the Information and Mathematical Models (DEIM), University of Palermo, Palermo, Italy; 2 Department of Mathematics, Linnaeus University, Växjö, Sweden; 3 National Institute for Nuclear Physics (INFN), Napoli, Italy; 4 Department of Social Studies, Linnaeus University, Växjö, Sweden; 5 Linnaeus University Center for Data Intensive Sciences & Applications (DISA@LNU), Växjö, Sweden; New York State Museum, UNITED STATES

## Abstract

The debate on the causes of conflict in human societies has deep roots. In particular, the extent of conflict in hunter-gatherer groups remains unclear. Some authors suggest that large-scale violence only arose with the spreading of agriculture and the building of complex societies. To shed light on this issue, we developed a model based on operatorial techniques simulating population-resource dynamics within a two-dimensional lattice, with humans and natural resources interacting in each cell of the lattice. The model outcomes under different conditions were compared with recently available demographic data for prehistoric South America. Only under conditions that include migration among cells and conflict was the model able to consistently reproduce the empirical data at a continental scale. We argue that the interplay between resource competition, migration, and conflict drove the population dynamics of South America after the colonization phase and before the introduction of agriculture. The relation between population and resources indeed emerged as a key factor leading to migration and conflict once the carrying capacity of the environment has been reached.

## Introduction

The debate on the causes of conflict in human societies has deep philosophical roots [1], with recent works even proposing it as one of the keys to understand human evolution [2]. However, the actual extent of the conflict in small hunter-gatherer groups remains unclear [3], with some authors suggesting that large-scale warfare and mass-killing only arose with the spreading of agriculture and the building of complex societies, as before resources were too sparse to make their conquest or defense meaningful [3–6]. Evidence supporting this view points out to the fact that, at least in some regions, the Neolithic revolution was accompanied by “unprecedented levels” of violence [7]. Even if evidence of widespread warfare also exists for previous periods [8–11], the question of whether the rate of conflict in pre-agricultural societies was sufficient to significantly affect the evolution of human populations on scales larger than the single group remains open [12].

In order to shed light on this issue, we developed a model based on operatorial techniques which simulates population-resource dynamics in a pre-agricultural context at the continental scale. The model outcomes under different conditions were compared with recently available demographic data for prehistoric South America [13]. Using as a proxy for population size the number of occupied archaeological sites and the probability density of summed calibrated radiocarbon dates (SCPDs), the authors reconstructed the spatio-temporal patterns of human population growth in South America, from 14 k to 2 k years ago. This period encompassed three phases: a first phase of rapid expansion from 14 k to 9 k years ago, which represents the continent colonization, followed by approximatively 4 k years of a dynamical equilibrium around a carrying capacity, with no net growth. Finally a new growth phase occurred, due to the rising of agriculture which allowed to sustain a larger population [13, 14].

Our research focuses on the pre-agriculture period and more specifically on the second phase, when the carrying capacity was reached: a condition which is simulated in our model. The demographic curve on that phase is characterized by sharp oscillations of large amplitude (see figure 3 in [13]), a behavior that resembles the dynamics of invasive species. Similar dynamics have indeed been observed in the field. However, the spatial scale of such observations is generally much smaller than the continental one [15]. Consequently, considering the whole South America as a single invaded site does not seem appropriate. From an ecological point of view, it could be better conceptualized as a mosaic of different ecosystems, in some cases separated by geographic barriers. Its population can hence be regarded as a meta-population split into different sub-populations, each of them occupying a patch of the mosaic [16]. This kind of representation appears especially suitable considering hunter-gatherers groups of the mid-Holocene, who normally lived within well defined territories whose size depended on the local ecological conditions [9, 12]. While in each patch of the mosaic we can expect oscillating dynamics between human and resource populations, there is no reason why these oscillations should be in phase on a larger scale, and empirical research confirmed that they often are not [17]. As a consequence, local oscillations should compensate each other, leading to a relatively smooth curve for the global population density.

This intuition was further confirmed by our model, where, consistently with the mosaic concept, South America is represented as a two-dimensional lattice, with humans and resources interacting in each cell of the lattice. The model produced demographic curves that indeed were oscillating at the local level (namely the single cell) but smooth at the global one (namely the whole lattice). The reconstructed curve for the population density in South America instead exhibits high peaks and dramatic drops, from a density of SCPDs less than 0.13 to almost 0.29 around a mean carrying capacity *K* ≈ 0.185, which cannot be entirely attributed to the calibration process [13]. This suggests that some coordinating mechanism must have been at work to keep in phase the local oscillations.

As a further step, we proposed a possible coordinating mechanism and implemented it into the model. More specifically, we argued that the interplay between resource competition, migration and conflict drove the population dynamics in South America after the colonization phase and before the introduction of agriculture. Unlike the no-migration case, under this second scenario, the model was able to fit remarkably well the reconstructed curve, strongly supporting the correctness of our interpretation. The rest of the paper is organized as follows. The next section illustrates our model, the subsequent one presents the results, and the last one discusses them and derives their general implications.

## Methods

### Model overview

We modeled South America as a two-dimensional lattice formed by *L*^2^ different cells. Each cell includes two interacting populations $P_{a}$ and $P_{b}$, respectively humans and natural resources. Being interested in a pre-agricultural situation, the latter population represents the only sustenance for humans. Starting from some initial conditions, populations evolve following a dynamics similar to the one of a predator-prey system, which from an ecological point of view is the most appropriate to represent a user-resource system with users depending on renewable biogenic resources [18, 19].

We explored two scenarios. In the first, no migration is allowed; in the second, humans are allowed to migrate to neighboring cells, while resources cannot diffuse across cells. Moreover, migration from a cell *α* to a cell *β* is assumed to depend on a variable *K*_*α*_, which reflects the local (i.e., in *α*) ratio between the densities of resources and humans. More specifically, humans migrate at a rate that increases for decreasing *K*_*α*_, simulating the fact that migration from *α* increases when local resources are low. After humans migrate from *α* to *β*, resources in *α* tend to recover, while human density in *β*, after an initial obvious increment, can subsequently decline. This produces a reduction of the total human populations in *α* and *β*, which we interpret as the outcome of increased mortality due to competition and/or conflict over local resources.

In order to reproduce population dynamics, we employed operatorial techniques commonly used in quantum mechanics and already adopted in the analysis of population dynamics and migration [20–22]. The details are presented in the following subsections.

### One-cell case

Let S be a system composed by two populations: the users $P_{a}$ (i.e. the humans), and the natural resources $P_{b}$. The system S can assume four basic states, while any other possible state can be obtained as a combination of these: *(i)* *φ*_0,0_, i.e. the *vacuum* of the system, corresponding to an almost complete absence of both humans and resources; *(ii)* *φ*_0,1_ (resp. *φ*_1,0_) corresponding to a low (resp. high) density of humans and abundance (resp. lack) of resources; *(iii)* *φ*_1,1_ abundance of both human and resources.

We associate the four basic states of the system with four independent mutually orthogonal vectors in a four-dimensional Hilbert space H endowed with the scalar product 〈⋅, ⋅〉, a procedure that follows the general framework described in details in Bagarello [20] and used in several contexts.

A convenient way to construct the vectors *φ*_*j*, *k*_ with *j*, *k* = 0,1 makes use of two fermionic operators, *a* and *b*, which together with their adjoints, *a*^†^ and *b*^†^, satisfy the canonical anticommutation relation (CAR):

$$\begin{array}{c} \left\{a,a^{†}\right\}=\left\{b,b^{†}\right\}={1\;1}_{4},\;a^{2}=b^{2}=0,\;\left\{a^{♯},b^{♯}\right\}=0, \end{array}$$ (1)

where {*x*, *y*} = *xy* + *yx* is the anticommutator of *x* and *y*, $a^{♯}$ can be both *a* or *a*^†^, and ${1\;1}_{4}$ is the identity operator in the Hilbert space $H=C^{4}$. We first introduce the vacuum vector *φ*_0,0_ which satisfies *aφ*_0,0_ = *bφ*_0,0_ = 0. The existence of a non zero vector *φ*_0,0_ with this property is a standard result in quantum mechanics [23]. The other vectors *φ*_*j*, *k*_ can be constructed from *φ*_0,0_ in the following way:

$$\begin{array}{c} \;\varphi_{1,0}:=a^{†}\varphi_{0,0},\;\varphi_{0,1}:=b^{†}\varphi_{0,0},\;\varphi_{1,1}:=a^{†}b^{†}\varphi_{0,0}. \end{array}$$

The set $F_{\varphi }=\left\{\varphi_{j,k},\;j,k=0,1\right\}$ is an orthonormal basis for H, so that a general vector Ψ of S can be expanded as $\Psi =\sum_{j,k=0}^{1}c_{j,k}\varphi_{j,k}$, with $\sum_{j,k=0}^{1}{\left|c_{j,k}\right|}^{2}=1$. By using a standard argument, we can interpret Ψ as a state where the probability to find S in the state *φ*_*j*, *k*_ is given by |*c*_*j*, *k*_|^2^.

We now introduce the number operators ${\hat{n}}^{\left(a\right)}=a^{†}a$ and ${\hat{n}}^{\left(b\right)}=b^{†}b$ of $P_{a}$ and $P_{b}$. Then:

$$\begin{array}{l} \begin{array}{ccc} {\hat{n}}^{\left(a\right)}\varphi_{n^{\left(a\right)},n^{\left(b\right)}} & = & n^{\left(a\right)}\varphi_{n^{\left(a\right)},n^{\left(b\right)}}, \end{array}\\ \begin{array}{ccc} {\hat{n}}^{\left(b\right)}\varphi_{n^{\left(a\right)},n^{\left(b\right)}} & = & n^{\left(b\right)}\varphi_{n^{\left(a\right)},n^{\left(b\right)}}, \end{array} \end{array}$$ (2)

where *n*^(*a*)^, *n*^(*b*)^ can be either 0 or 1. It holds:

$$\begin{array}{c} {\hat{n}}^{\left(a\right)}\Psi =\sum_{k=0}^{1}c_{1,k}\varphi_{1,k},\;{\hat{n}}^{\left(b\right)}\Psi =\sum_{j=0}^{1}c_{j,1}\varphi_{j,1}, \end{array}$$

and the mean values, or densities, *n*^(*a*), (*b*)^ of the operators ${\hat{n}}^{\left(a),(b\right)}$ over the state Ψ are defined as $n^{\left(a),(b\right)}=\langle \Psi ,{\hat{n}}^{\left(a),(b\right)}\Psi \rangle$, leading to the following expressions:

$$\begin{array}{c} n^{\left(a\right)}=\sum_{k=0}^{1}|c_{1,k}{|}^{2},\;n^{\left(b\right)}=\sum_{j=0}^{1}{\left|c_{j,1}\right|}^{2}. \end{array}$$ (3)

From a biological point of view, *n*^(*a*)^ (resp. *n*^(*b*)^) is interpreted as the density for the population $P_{a}$ (resp. $P_{b}$) associated with the state Ψ. Both *n*^(*a*)^, *n*^(*b*)^ are real numbers between 0 and 1. For instance, if at *t* = 0 *n*^(*a*)^ = 0, it means that Ψ is only a combination of *φ*_0,0_ and *φ*_0,1_ corresponding to the (almost complete) absence of humans (zero density for $P_{a}$). Conversely, if *n*^(*a*)^ = 1 then Ψ is a combination of *φ*_1,0_ and *φ*_1,1_ corresponding to the maximum density for humans.

### 2D case

A square lattice divided in *L*^2^ cells is introduced in the 2D case. In each cell the two populations $P_{a}$ and $P_{b}$ interact as in the previous case.

In each cell *α* = 1, ⋯, *L*^2^, we introduce the fermionic operators *a*_*α*_, $a_{\alpha }^{†}$ and the related number operators ${\hat{n}}_{\alpha }^{\left(a\right)}=a_{\alpha }^{†}a_{\alpha }$ for $P_{a}$ and *b*_*α*_, $b_{\alpha }^{†}$ and ${\hat{n}}_{\alpha }^{\left(b\right)}=b_{\alpha }^{†}b_{\alpha }$ for $P_{b}$. As before, we suppose that these operators satisfy the standard CAR anticommutation rules:

$$\begin{array}{ccc} \left\{a_{\alpha },a_{\beta }^{†}\right\} & = & \left\{b_{\alpha },b_{\beta }^{†}\right\}=\delta_{\alpha \beta }1\;1,\;\forall \alpha ,\beta \end{array}$$ (4)

$$\begin{array}{ccc} \left\{a_{\alpha }^{♯},a_{\beta }^{♯}\right\} & = & \left\{b_{\alpha }^{♯},b_{\beta }^{♯}\right\}=\left\{a_{\alpha }^{♯},b_{\beta }^{♯}\right\}=0,\;\forall \alpha \neq \beta . \end{array}$$ (5)

Moreover $a_{\alpha }^{2}=b_{\alpha }^{2}=0$ holds for all *α*. In Eq (4) 11 is the identity operator on the Hilbert space H, with $\dim\left(H\right)=4^{L^{2}}$, endowed with the scalar product 〈⋅, ⋅〉.

Extending what we have done before, we first introduce the *vacuum* vector of the system $\varphi_{{\vec{0}}_{a},{\vec{0}}_{b}}$, where ${\vec{0}}_{a}={\vec{0}}_{b}=\left(0,0,\cdots ,0\right)$ are two *L*^2^-dimensional vectors. The *vacuum* is annihilated by all the operators *a*_*α*_, *b*_*α*_, i.e.,

$$\begin{array}{c} a_{\alpha }\varphi_{{\vec{0}}_{a},{\vec{0}}_{b}}=b_{\alpha }\varphi_{{\vec{0}}_{a},{\vec{0}}_{b}}=0,\;\forall \alpha =1,..,L^{2}. \end{array}$$

We then construct the states of the basis of H by acting with the operators $a_{\alpha }^{†},b_{\alpha }^{†}$ over $\varphi_{{\vec{0}}_{a},{\vec{0}}_{b}}$,

$$\begin{array}{c} \varphi_{{\vec{m}}_{a},{\vec{m}}_{b}}={\left(a_{1}^{†}\right)}^{m_{a}\left(1\right)}\cdots {\left(a_{L^{2}}^{†}\right)}^{m_{a}\left(L^{2}\right)}{\left(b_{1}^{†}\right)}^{m_{b}\left(1\right)}\cdots {\left(b_{L^{2}}^{†}\right)}^{m_{b}\left(L^{2}\right)}\varphi_{{\vec{m}}_{a},{\vec{m}}_{b}}, \end{array}$$ (6)

where ${\vec{m}}_{a}=(m_{a}\left(1\right),\cdots ,m_{a}\left(L^{2}\right))$, ${\vec{m}}_{b}=\left(m_{b}\left(1\right),\cdots ,m_{b}\left(L^{2}\right)\right)$ are all the possible *L*^2^-dimensional vectors whose entries are only 0 or 1. In fact, ${a_{\alpha }^{†}}^{2}={b_{\alpha }^{†}}^{2}=0$, any other choice would destroy the state. Notice that we can construct at most 2^*L*^2^^ vectors ${\vec{m}}_{a}$ and 2^*L*^2^^ vectors ${\vec{m}}_{b}$, so that we can build 4^*L*^2^^ possible pairs $\left({\vec{m}}_{a},{\vec{m}}_{b}\right)$.

The set $F_{\varphi }$ of all the vectors obtained by this construction forms an orthonormal basis of H. Moreover:

$$\begin{array}{ccc} {\hat{n}}_{\alpha }^{\left(a\right)}\varphi_{{\vec{m}}_{a},{\vec{m}}_{b}} & = & m_{a}\left(\alpha \right)\varphi_{{\vec{m}}_{a},{\vec{m}}_{b}}, \end{array}$$ (7)

$$\begin{array}{ccc} {\hat{n}}_{\alpha }^{\left(b\right)}\varphi_{{\vec{m}}_{a},{\vec{m}}_{b}} & = & m_{b}\left(\alpha \right)\varphi_{{\vec{m}}_{a},{\vec{m}}_{b}}, \end{array}$$ (8)

for all *α* = 1, ⋯, *L*^2^. For more details on CAR, we refer to Roman [23].

The vectors $\varphi_{{\vec{m}}_{a},{\vec{m}}_{b}}$ can now be interpreted similarly to *φ*_*j*, *k*_ in the previous section. The vacuum $\varphi_{{\vec{0}}_{a},{\vec{0}}_{b}}$, for instance, describes a situation where all the lattice cells hold few of both humans and resources. The vector $\varphi_{{\vec{m}}_{a},{\vec{m}}_{b}}$ with ${\vec{m}}_{a}=(1,0,0\cdots ,0)$ and ${\vec{m}}_{b}=\left(0,0,\cdots ,0,1\right)$ describes the same situation, except that there is a large amount of humans in the first cell and of resources in the last one.

A generic state Ψ(0) of the system can be written as a linear combination of the elements in $F_{\varphi }$:

$$\begin{array}{c} \Psi \left(0\right)=\sum_{{\vec{m}}_{a},{\vec{m}}_{b}}c_{{\vec{m}}_{a},{\vec{m}}_{b}}\varphi_{{\vec{m}}_{a},{\vec{m}}_{b}}, \end{array}$$ (9)

where $c_{{\vec{m}}_{a},{\vec{m}}_{b}}$ are complex scalars such that $\sum_{{\vec{m}}_{a},{\vec{m}}_{b}}{\left|c_{{\vec{m}}_{a},{\vec{m}}_{b}}\right|}^{2}=1$.

From Eqs (7) and (8), the $\varphi_{{\vec{m}}_{a},{\vec{m}}_{b}}$ are eigenstates of the number operators and the densities of $P_{a}$ and $P_{b}$ in *α* are simply their related eigenvalues. This is true at the initial time *t* = 0. At a later time, we need to compute the mean values over the state Ψ(*t*) describing the system S at time *t*:

$$\begin{array}{ccc} n_{\alpha }^{\left(a\right)}\left(t\right) & = & \left\langle \Psi \left(t\right),{\hat{n}}_{\alpha }^{\left(a\right)}\Psi \left(t\right)\right\rangle ={∥a_{\alpha }\Psi \left(t\right)∥}^{2}, \end{array}$$ (10)

$$\begin{array}{ccc} n_{\alpha }^{\left(b\right)}\left(t\right) & = & \left\langle \Psi \left(t\right),{\hat{n}}_{\alpha }^{\left(b\right)}\Psi \left(t\right)\right\rangle ={∥b_{\alpha }\Psi \left(t\right)∥}^{2}, \end{array}$$ (11)

As already stated (see also [20]), these mean values are phenomenologically interpreted as densities of humans and resources in the cell *α*.

### Evolution of the system

To determine the densities Eqs (10) and (11), we need first to compute the time evolution of the state of the system Ψ(*t*). If $\Psi \left(0\right)=\Psi_{0}=\sum_{{\vec{m}}_{a},{\vec{m}}_{b}}c_{{\vec{m}}_{a},{\vec{m}}_{b}}\varphi_{{\vec{m}}_{a},{\vec{m}}_{b}}$, then Ψ(*t*) can be written as $\Psi \left(t\right)=\sum_{{\vec{m}}_{a},{\vec{m}}_{b}}c_{{\vec{m}}_{a},{\vec{m}}_{b}}\left(t\right)\varphi_{{\vec{m}}_{a},{\vec{m}}_{b}}$.

The time-depending functions $c_{{\vec{m}}_{a},{\vec{m}}_{b}}\left(t\right)$ are obtained through the Schrödinger equation $i\frac{\partial \Psi (t)}{\partial t}=H\;\Psi \left(t\right)$, where *H* is the Hamiltonian operator describing the dynamics of humans and resources. To make the model more realistic, we assume that *H* explicitly depends on time and on the local densities of $P_{a}$ and $P_{b}$. Using the orthogonality conditions of the basis vectors $\varphi_{{\vec{m}}_{a},{\vec{m}}_{b}}$, we obtain the following ordinary differential equation (ODE) system for the coefficients $c_{{\vec{m}}_{a},{\vec{m}}_{b}}\left(t\right)$

$$\begin{array}{c} i\frac{\partial c_{{\vec{m}}_{a},{\vec{m}}_{b}}\left(t\right)}{\partial t}=\left\langle \varphi_{{\vec{m}}_{a},{\vec{m}}_{b}},H\Psi \left(t\right)\right\rangle , \end{array}$$ (12)

which, when solved, returns the densities of humans and resources in *α*:

$$\begin{array}{l} n_{\alpha }^{\left(a\right)}\left(t\right)=\sum_{\begin{array}{c} {\vec{m}}_{a},{\vec{m}}_{b}\\ m_{a}\left(\alpha \right)=1 \end{array}}{\left|c_{{\vec{m}}_{a},{\vec{m}}_{b}}\left(t\right)\right|}^{2},\\ n_{\alpha }^{\left(b\right)}\left(t\right)=\sum_{\begin{array}{c} {\vec{m}}_{a},{\vec{m}}_{b}\\ m_{b}\left(\alpha \right)=1 \end{array}}{\left|c_{{\vec{m}}_{a},{\vec{m}}_{b}}\left(t\right)\right|}^{2}. \end{array}$$ (13)

Note that the above general construction implies that both $n_{\alpha }^{\left(a\right)}\left(0\right)\leq {∥\Psi \left(0\right)∥}^{2}=1$ and $n_{\alpha }^{\left(b\right)}\left(0\right)\leq {∥\Psi \left(0\right)∥}^{2}=1$ at *t* = 0. As we will see later (see Eq (20)), this condition is preserved for all time, even in the current setting where the Hamiltonian is not purely quadratic and time independent.

It remains now to explicitly define the self—adjoint Hamiltonian of the system. The human-resource interaction is ruled in each cell *α* by the operator *H*_*α*_ while the migration is ruled by the operator *H*_*M*_, so that the full Hamiltonian is given by: *H* = (∑_*α*_ *H*_*α*_)+*H*_*M*_, where the last term is clearly zero in the first scenario, i.e., in absence of migration. A natural choice for *H*_*α*_ and *H*_*M*_ is:

$$\begin{array}{ccc} H_{\alpha } & = & \omega_{\alpha }^{a}\left(t\right)a_{\alpha }^{†}a_{\alpha }+\omega_{\alpha }^{b}\left(t\right)b_{\alpha }^{†}b_{\alpha }+\lambda_{\alpha }\left(t\right)\left(a_{\alpha }^{†}b_{\alpha }+b_{\alpha }^{†}a_{\alpha }\right), \end{array}$$ (14)

$$\begin{array}{ccc} H_{M} & = & \sum_{\alpha \neq \beta }p_{\alpha ,\beta }\gamma_{\alpha ,\beta }\left(t\right)a_{\alpha }a_{\beta }^{†}, \end{array}$$ (15)

where *p*_*α*, *β*_ ≠ 0 if *α* and *β* are neighboring cells, and *p*_*α*, *β*_ = 0 otherwise.

The meaning of the terms *H*_*α*_, *H*_*M*_ has been explained in details and discussed in [21] and [20]. Here we simply recall that the terms in $\omega_{\alpha }^{a}\left(t\right)a_{\alpha }^{†}a_{\alpha }$ and $\omega_{\alpha }^{b}\left(t\right)b_{\alpha }^{†}b_{\alpha }$ are related to a sort of (time-dependent) *inertia* of the populations within the system, because increasing the values $\omega_{\alpha }^{a,b}\left(t\right)$ leads to a more static behavior of the associated populations keeping the densities close to their initial values. The terms $\lambda_{\alpha }\left(t\right)(a_{\alpha }^{†}b_{\alpha }+b_{\alpha }^{†}a_{\alpha })$ are the interaction parts of the Hamiltonian, where the functions *λ*_*α*_(*t*) measure the strength of the interaction between $P_{a}$ and $P_{b}$ in *α*. This interaction leads to a sort of predator-prey dynamics, with the term $a_{\alpha }^{†}b_{\alpha }$ that increases the density of $P_{a}$ whereas the density of $P_{b}$ decreases. The hermitian conjugate term $a_{\alpha }^{†}b_{\alpha }$ induces an opposite effect. The terms $a_{\alpha }a_{\beta }^{†}$ in *H*_*M*_ rule the migration of humans between *α* and *β*, because $a_{\alpha }a_{\beta }^{†}$ increases the density of humans in *β* while decreasing the density in *α*. Of course, such a migration is only possible for non zero values of *p*_*α*, *β*_.

Finally, we have to define the analytic expression for the functions $\omega_{\alpha }^{a,b}\left(t\right)$, *λ*_*α*_(*t*), and *γ*_*α*, *β*_(*t*). When migration is allowed, we assume that humans migrate from a cell *α* to a neighboring one only if the local human/resource ratio $K_{\alpha }\left(t\right)=n_{\alpha }^{\left(b\right)}\left(t\right)/n_{\alpha }^{\left(a\right)}\left(t\right)$ is low enough, with migration that intensifies when *K*_*α*_(*t*) decreases. Moreover, natural resources tend to proliferate in a given cell in case of low human density, namely if *K*_*α*_(*t*) is large, otherwise they tend to exhaust.

In accordance to such assumptions, we chose the following functions:

$$\begin{array}{ccc} \omega_{\alpha }^{a}\left(t\right) & = & \sigma_{\alpha }{\left(\exp^{-{\left(K_{\alpha }{\left(t\right)}^{-1}/\tau_{\alpha }\right)}^{2}}K_{\alpha }\left(t\right)\right)}^{1/2}, \end{array}$$ (16)

$$\begin{array}{ccc} \omega_{\alpha }^{b}\left(t\right) & = & \sigma_{\alpha }{\left(\exp^{-{\left(K_{\alpha }\left(t\right)/\tau_{\alpha }\right)}^{2}}K_{\alpha }{\left(t\right)}^{-1}\right)}^{1/2}, \end{array}$$ (17)

$$\begin{array}{ccc} \lambda_{\alpha }\left(t\right) & = & \omega_{\alpha }^{a}\left(t\right)+\omega_{\alpha }^{b}\left(t\right)+\mu_{\alpha }, \end{array}$$ (18)

$$\begin{array}{ccc} \gamma_{\alpha ,\beta }\left(t\right) & = & \omega_{\alpha }^{b}\left(t\right)+\omega_{\beta }^{b}\left(t\right), \end{array}$$ (19)

where *σ*_*α*_, *τ*_*α*_, *μ*_*α*_ are real parameters. From Eqs (16)–(19), it follows that the higher the value of *K*_*α*_(*t*), the higher (resp. the smaller) the value of $\omega_{\alpha }^{a}\left(t\right)$ (resp. $\omega_{\alpha }^{b}\left(t\right)$). As a consequence, when *K*_*α*_(*t*) is high the migratory effects are mainly given by the contribution $\omega_{\beta }^{b}\left(t\right)$ in *γ*_*α*, *β*_(*t*). For instance, when in *β* the ratio resources/humans is low then the migration occurs mainly from *β* to *α*. The function *λ*_*α*_(*t*) in Eq (18) increases for high values of $\omega_{\alpha }^{a}\left(t\right)$ or $\omega_{\alpha }^{b}\left(t\right)$, i.e., when *K*_*α*_(*t*) is either high or low. This implies that, when human density is low in a cell, it grows at the expense of the resources and vice versa.

The fixed term *μ*_*α*_ has been introduced to guarantee a minimal predator-prey dynamics even when both $\omega_{\alpha }^{a}\left(t\right)$ an $\omega_{\alpha }^{b}\left(t\right)$ are close to zero. Finally, the values of *σ*_*α*_, *τ*_*α*_ tune how strongly *K*_*α*_(*t*) affects the system dynamics. In particular, by decreasing *τ*_*α*_ we limit the interaction effect only to the case of very high or very low values of *K*_*α*_(*t*), while increasing *σ*_*α*_ strengthens it.

Since *H* = *H*^†^ a general argument shows that

$$\begin{array}{c} n^{\left(a\right)}\left(t\right)+n^{\left(b\right)}\left(t\right)=\sum_{\alpha }\left(n_{\alpha }^{\left(a\right)}\left(t\right)+n_{\alpha }^{\left(b\right)}\left(t\right)\right)=n^{\left(a\right)}\left(0\right)+n^{\left(b\right)}\left(0\right), \end{array}$$ (20)

so that, if the global population density increases, the global resources density decreases and vice-versa. The quantity $K=\frac{n^{\left(a\right)}\left(0\right)+n^{\left(b\right)}\left(0\right)}{2}$ can be interpreted as a sort of carrying capacity, since the various terms in the Hamiltonian work to reduce *n*^(*a*)^(*t*) (and to increase *n*^(*b*)^(*t*)) when *n*^(*a*)^(*t*) > *K*, and vice-versa.

The values of the parameters used in our numerical simulations to reproduce the empirical data were *σ*_*α*_ = 12.5, *τ*_*α*_ = 0.35, *μ*_*α*_ = 0.25, *p*_*α*, *β*_ = 1, ∀*α*, *β*. We further fixed *K* = 0.5. The coefficients in Eq (9) were chosen so that the initial human density $n_{\alpha }^{\left(a\right)}\left(0\right)$ in each cell randomly differs at most by 10% from *K*/*L*^2^, and with the further global condition *n*^(*a*)^(0) = *K*. The rationale for this choice was to reproduce the phase when the carrying capacity was reached in the continent. The resources densities were set to $n_{\alpha }^{\left(b\right)}\left(0\right)=(2K/L^{2}-n_{\alpha }^{\left(a\right)}\left(0\right))$.

Notice that $\omega_{\alpha }^{a}\left(t\right)$ or $\omega_{\alpha }^{b}\left(t\right)$ can theoretically blow-up when $n_{\alpha }^{\left(a\right)}=0$ or $n_{\alpha }^{\left(b\right)}=0$, i.e., when human or resource local density become zero. However, these conditions never occurred in the simulations, as our initial conditions ensured non zero local densities in each cell. Indeed, even if at some time a local density approaches zero, the interaction and migration terms in *H* work to avoid any further decreasing. The Matlab code used for the simulation is presented as supporting information.

## Results

### No-migration scenario

**Fig 1:**
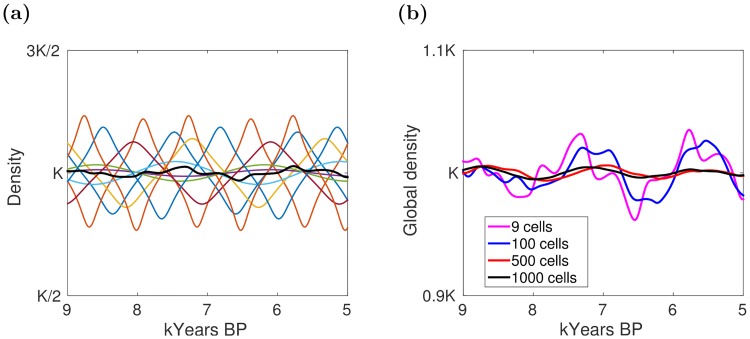
Model dynamics in absence of migration. *K* represents the system carrying capacity, i.e., the maximum human population density that the system can sustain indefinitely. (a) Single-cell human population density (colored curves) and global human population density (black thicker curve) in a nine-cell system. Single-cell densities were rescaled by a factor of 9 for comparison purposes. (b) Global human population density in systems composed by different number of cells.

We implemented the model described above with a number of cells varying from 9 to 1000. Although in each cell we observed oscillating dynamics, when considering the global population density—i.e., the density of the population in the whole lattice—the curve became smoother as oscillations compensated each other (Fig 1a). In addition, we observed that this smoothing effect increased with the number of cells included in the model (Fig 1b). As a result, the global density curve became dramatically different from the one for South America between 9 and 5.5 k years BP (see Fig 3b in [13]) suggesting that some coordination mechanism, able to keep in phase the local oscillations, was at work.

### Migration scenario

In the second scenario, migration was allowed, i.e., humans could move from their cell to a neighboring one, with a possible subsequent decline of population due to increased competition over natural resources (see the Methods section). The model parameters were set to fit the empirical data presented in [13]. Due to computational constraints, simulations of the second scenario are shown here only for an *L*^2^ lattice with *L* = 3. Slightly larger lattices were also considered but no significant difference was observed.

**Fig 2:**
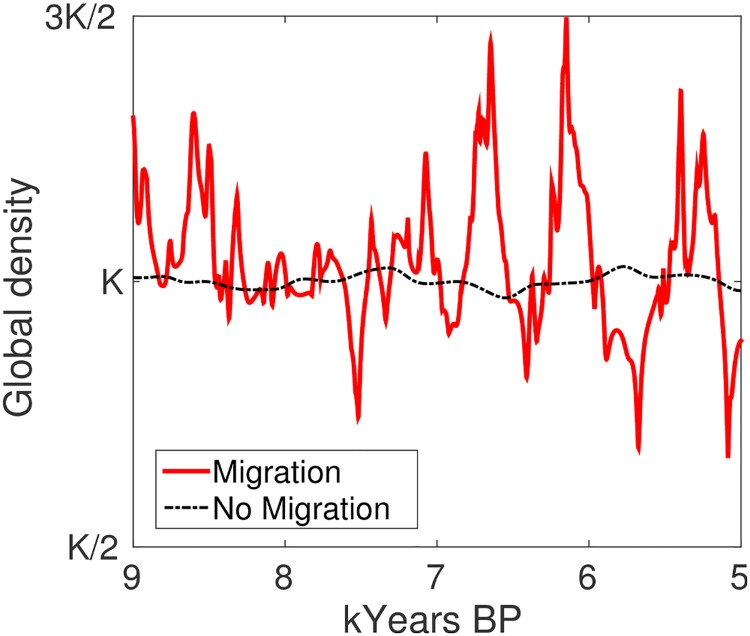
Model dynamics in a nine-cell system with and without migration. The no-migration scenario produced global density fluctuations in the [0.96*K*, 1.03*K*] interval, the migration scenario in the [0.66*K*, 1.50*K*] one.

**Fig 3:**
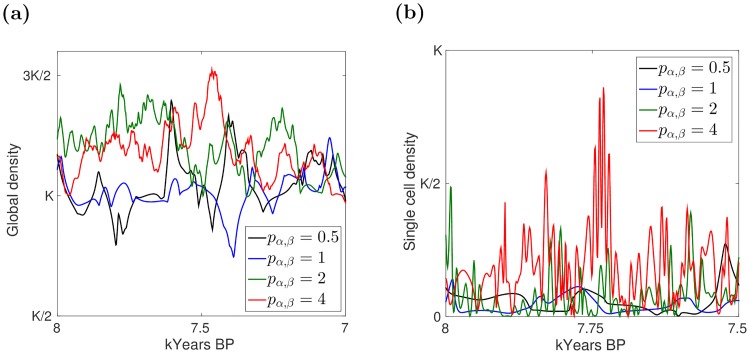
Model dynamics for different values of the migration parameters *p*_*α*, *β*_. (a) Global (whole nine-cell lattice) human population density. (b) Local (single cell) human population density, with the central cell selected as example. Note that, to improve visualization, the panels do not show the whole 9–5 kYears BP time interval.

At the scale of the single cell, we observed oscillating dynamics as in the previous scenario, albeit sharper. However, unlike the previous case, oscillations did not compensate each others at the global level and the global population density curve exhibited peaks and drops consistent with the ones empirically estimated for South America (Fig 2). Specifically, we compared the amplitude with respect to the carrying capacity of the oscillations in the empirical curve and in the one produced by the model. This comparison highlighted that under the migration scenario the curves have a very similar behavior: in our model the global population density oscillations fell in the the [0.66*K*, 1.50*K*] interval, while in Fig 3b by Goldberg *et al*. they fell in the [0.70*K*, 1.57*K*] interval [13]. In addition, we considered the most relevant peaks and valleys (i.e., the ones almost reaching the interval limits) of both curves, and their number turned to be very similar.

In order to test the model robustness, we performed a sensitivity analysis using a wide range of parameter configurations. Under no condition we were able to reproduce the empirically estimated oscillations in absence of migration and conflict, which hence emerged in our model as a crucial mechanism able both to exacerbate the local oscillations and to keep them synchronized at the global level.

How the various parameters affect the Hamiltonian Eq (15) is well understood from [20], and similar conclusions hold for the present model, with some differences due to the nonlinear parameters that have been introduced here (see the Sensitivity analysis section in the Supporting information). For instance, increasing the value of $\omega_{\alpha }^{a}\left(t\right)$ produces a stronger inertia for humans in cell *α*, with their density exhibiting fewer and smaller oscillations. Changing the parameters *p*_*α*, *β*_, which tunes migration, we obtained different oscillatory behaviors of the population density. In particular, increasing *p*_*α*, *β*_ induced the formation of sharp oscillations in both the global and local density curves, even if this is especially evident at the local (single cell) level (Fig 3). Further details on the sensitivity analysis are included as Supporting information.

## Conclusions

Recent data suggested that pre-agricultural human populations were subjected to significant oscillations on scales larger than the single group and reaching the continental level. Although part of the empirically-estimated oscillations for South America may be an effect of the calibration process, statistical analysis proved them to be significantly different from a random dynamics [13]. Our operator-based model showed that the simple aggregation of local fluctuations, in absence of a more general coordination mechanism, could not reproduce the observed dynamics. A mechanism that our model instead proved to be sufficient to explain the observed dynamics was the synergistic work of migration and conflict.

The picture that can be derived is that, in case of local resource overuse, mobile hunter-gathered groups tried to move to neighboring territories. If these were already occupied, as in South America after the colonization phase, migration likely led to conflict. Both scarcity and conflict hence had the potential to spread in growing circles, with a snowball effect that could reach even the continental scale in a relatively short time.

A major advantage of our interpretation lies on the fact that it is consistent with what we know about hunter-gatherer groups which, as shown by anthropological research, presented significant levels of violence fundamentally caused by competition over resources [9, 10]. In particular, our results are consistent with recent findings showing that reduced environmental productivity is a strong predictor of lethal aggression among prehistoric groups in Central California [24].

The main limit of our work lies in the fact that it has only been checked against a specific phase within a single dataset. New datasets could be used in the future for further testing. North America and Australia look as especially interesting cases as they both share with South America a story of colonization by humans of previously unoccupied territories. Moreover, at the current stage of the research, we only modelled a period of about 4000 years, which is significantly shorter than the time-range of Goldberg and colleagues’ dataset. In future steps, both the colonization phase and the expansion of the carrying capacity linked to the development of agriculture could be included in the model.

Despite these limitations, the current version of our model was already able to shed light on the long enduring debate about the roots of conflict for humans societies [12]. It has formerly been proposed that, due to their low density and the few possessions they owned, conflict among hunter-gatherer groups was limited, if not absent [3–6]. This “peaceful noble savage” view has already been challenged elsewhere [8–11, 24], and our model showed that it does perhaps not apply to prehistoric South America as well. More than the lifestyle (hunter-gatherer vs. more complex societies), the relation between population and resources appears to be a key factor eventually leading to migration and conflict once the carrying capacity of a given environment has been reached.

The progressive introduction of agriculture in South America allowed for an increase in the carrying capacity, leading to a new population expansion phase between 5.5 and 2 k years ago [13]. However, also the technological expansion of the possibilities of the natural environment has its limits, and new resource conflicts are likely to arise when they are reached. As planetary boundaries are likely to become a major concern in the near future [25, 26], with a possible increase of environment-related conflict [27–29], the lessons that can be derived from the modeling of prehistoric large-scale demographic dynamics could provide valuable insights also for today’s world.

## Supporting information

S1 Appendix Sensitivity analysis. This appendix presents an extended sensitivity analysis on the model.
(PDF)

S2 Appendix Model code. This appendix presents the Matlab code used for the numerical simulation.
(PDF)

S1 Fig Model dynamics for different values of *μ*_*α*_. (a) Global (whole nine-cell lattice) human population density in the migration scenario. (b) Global human population density in the no-migration scenario. (c) Local (single cell) human population density in the migration scenario, with the central cell selected as example. (d) Local human population density in the no-migration scenario.
(TIF)

S2 Fig Model dynamics for different values of *τ*_*α*_. (a) Global (whole nine-cell lattice) human population density in the migration scenario. (b) Global human population density in the no-migration scenario. (c) Local (single cell) human population density in the migration scenario, with the central cell selected as example. (d) Local human population density in the no-migration scenario.
(TIF)

S3 Fig Model dynamics for different values of the migration parameter *τ*_*α*_. (a) Global human population density in the migration scenario. (b) Global human population density in the no-migration scenario.
(TIF)
