# Supplementary material for: Large-scale effects of migration and conflict in pre-agricultural groups: Insights from a dynamic model
Source: PLoS One. 2017 Mar 8;12(3):e0172262. doi: 10.1371/journal.pone.0172262 (PMC5342208; doi:10.1371/journal.pone.0172262)
Supplement: S1 Appendix — This appendix presents an extended sensitivity analysis on the model. (PDF) [file pone.0172262.s001.pdf]

Extended Sensitivity Analysis for: Large-scale effects of migration and conflict in pre-agricultural groups: Insights from a dynamic model

Francesco Gargano[1], Lucia Tamburino[2], Fabio Bagarello[1,3], Giangiacomo Bravo[4,5,*]

1 Department of Energy, Engineering of the Information and Mathematical Models (DEIM), University of Palermo, Palermo, Italy
2 Department of Mathematics, Linnaeus University, Växjö, Sweden
3 National Institute for Nuclear Physics (INFN), Napoli, Italy
4 Department of Social Studies, Linnaeus University, Växjö, Sweden
5 Linnaeus University Center for Data Intensive Sciences & Applications (DISA@LNU), Växjö, Sweden

* giangiacomo.bravo@lnu.se

Sensitivity Analysis

This section presents a sensitivity analysis on the parameters τ_α and μ_α of the Hamiltonian, see (16)–(19). It is clear that the parameter σ_α simply acts as a re-scaling of $\omega_\alpha^{a,b}$, $\gamma_{\alpha,\beta}$ and the ratio $(\omega_\alpha^a + \omega_\alpha^b)/\mu_\alpha$ in (16). Therefore, the global effect of modifying σ_α essentially is a time re-scaling. For instance, increasing σ_α we speed up the dynamics so that the density curves exhibits rapid oscillations concentrated on a smaller time range.

As explained in the main text, $\mu_\alpha > 0$ tunes the predator-prey effect in the dynamics between humans and resources. Figure S1 shows the model outcome for different values of μ_α. In the migration scenario, as expected, oscillations become sharper and wider for larger values of μ_α (Fig. S1a). This is especially evident in the single cell plot where no compensation effect due to summing does occur (Fig. S1c). In the no-migration scenario ($p_{\alpha,\beta} = 0, \forall \alpha, \beta$), even for high values of μ_α the global densities always remain close to the carrying capacity K (Fig. S1b).

Figure S2 shows the model outcome for different values of τ_α, which tunes how strongly K_α affects the system dynamics. Specifically, by decreasing τ_α, human-resource interaction and migration are limited to the cases of very high or very low values of K_α. If K_α initially is close to the equilibrium (i.e., $K_\alpha \approx 1$, as assumed in the simulations), interaction and migration are weakened. This can be observed in Figure S2, where for $\tau_\alpha = 0.175$ the human density curves only show minor variations in both scenarios. For higher values of τ_α, sharper oscillations arise even in the $K_\alpha \approx 1$ case but only in the migration scenario (as before, more evidently in the single cell plot). In the no-migration scenario, the only observed effect is an increase of the frequency of the small oscillations.

Finally, it is worth noting that the non-linearity of the Hamiltonian parameters (see 16–19) may induce chaotic dynamics in the system even for small variations of the parameters. For instance, Figure S3 shows the global density curves for small variations of τ_α from the initial time (in our time unit 10 KYears BP). In the migration scenario, increasing τ_α, the first peak is slightly anticipated but the curves almost overlap. However, over time they dramatically diverge (Fig. S3a). In the no-migration scenario, this chaotic behavior is greatly mitigated since the global density curves always remain close to the carrying capacity K.

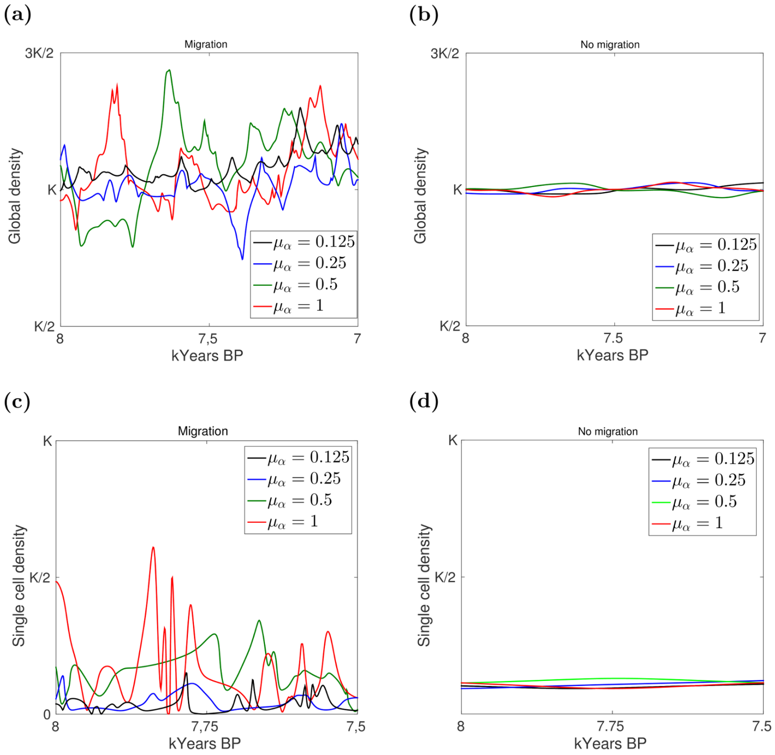

Fig S1. Model dynamics for different values of μ_α. (a) Global (whole nine-cell lattice) human population density in the migration scenario. (b) Global human population density in the no-migration scenario. (c) Local (single cell) human population density in the migration scenario, with the central cell selected as example. (d) Local human population density in the no-migration scenario.

Overall, the sensitivity analysis confirmed the crucial role of migration as the only way to reproduce the empirical oscillations observed in [1].

References

1. Goldberg A, Mychajliw AM, Hadly EA. Post-invasion demography of prehistoric humans in South America. Nature. 2016;532(7598):232–235. doi:10.1038/nature17176.

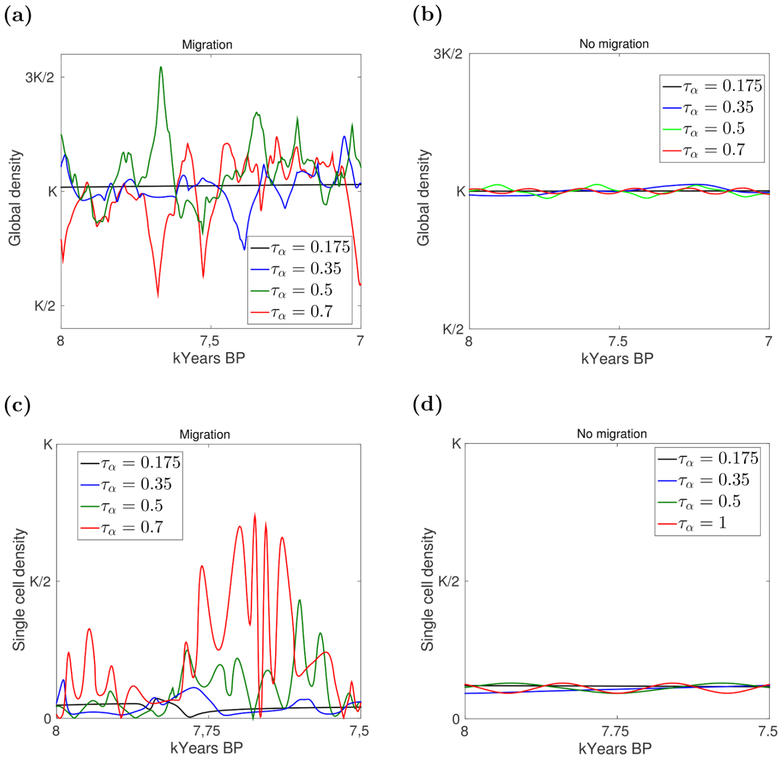

Fig S2. Model dynamics for different values of τ_α. (a) Global (whole nine-cell lattice) human population density in the migration scenario. (b) Global human population density in the no-migration scenario. (c) Local (single cell) human population density in the migration scenario, with the central cell selected as example. (d) Local human population density in the no-migration scenario.

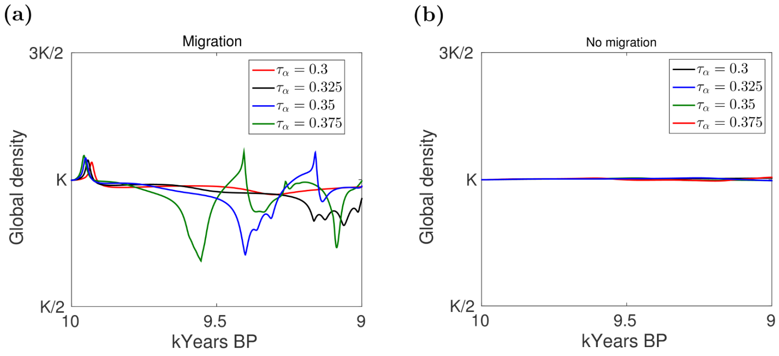

Fig S3. Model dynamics for different values of the migration parameters τ_α. (a) Global human population density in the migration scenario. (b) Global human population density in the no-migration scenario.
