# Supplementary material for: Large-scale effects of migration and conflict in pre-agricultural groups: Insights from a dynamic model
Source: PLoS One. 2017 Mar 8;12(3):e0172262. doi: 10.1371/journal.pone.0172262 (PMC5342208; doi:10.1371/journal.pone.0172262)
Supplement: S2 Appendix — This appendix presents the Matlab code used for the numerical simulation. (PDF) [file pone.0172262.s002.pdf]

Model Code for: Large-scale effects of migration and conflict in pre-agricultural groups: Insights from a dynamic model

Francesco Gargano[1], Lucia Tamburino[2], Fabio Bagarello[1,3], Giangiacomo Bravo[4,5,*]

1 Department of Energy, Engineering of the Information and Mathematical Models (DEIM), University of Palermo, Palermo, Italy
2 Department of Mathematics, Linnaeus University, Växjö, Sweden
3 National Institute for Nuclear Physics (INFN), Napoli, Italy
4 Department of Social Studies, Linnaeus University, Växjö, Sweden
5 Linnaeus University Center for Data Intensive Sciences & Applications (DISA@LNU), Växjö, Sweden

* giangiacomo.bravo@lnu.se

Model code

This section presents the Matlab code used for the numerical simulation. The code was originally divided in three files, namely *MAIN.m*, *init.m*, and *shroedingerevol.m*.

Main (MAIN.m file)

```
%%NUMBER OF INITIAL CELL%%
L=9:
%%%%%%%%%%%%%%%%%%%%%%%%

%% TIME INTERVAL %%
tspan=[0:0.001:1];
%%%%%%%%%%%%%%%%%%%%%%%%

%% LAUNCH PROCEDURE FOR STORING MATRIX AND VECTORS %%
init;
%%%%%%%%%%%%%%%%%%%%%%%%

%% LAUNCH SHROEDINGER EVOLUTION%%
[psi,T,NH,NR]=shroedingerevol(L,p,phiin,MatrixR,HH,HR,Hwh,Hwr,tspan);

%OUTPUT:
% psi:=the evolved vector psi for all time in tspan
% T:=the tspan
% NH:= human densities in the cells at each time
% NR:= densities of resources in the cells at each time
%%%%%%%%%%%%%%%%%%%%%%%%
```

Initialization (init.m file)

```
%%Initial densities for Humans (dens_initH) and Resources (dens_initR)

for j=1:L
   dens_initH(j)=rand(1);
   dens_initR(j)=1-dens_initH(1);
end
```

```
%Ground state
phi0=1;
phi0(4^L)=0;
phi0=phi0';

%Oauli's Matrices
sp=[0 1;0 0];
sz=[1 0;0 -1];
Id=[1 0;0 1];

% Fermionic Annihilation operators
p{2*L}=0;
for N=1:2*L;
  p{N}=sparse(sp);
  for n=N:2*L-1
    p{N}=kron(Id,p{N});
  end
  for n=1:N-1
    p{N}=kron(p{N},sz);
  end
end

%Initial condition: phiin
phiin=phi0;
phiin=sqrt(dens_initH(1))*p{1}'*phi0;
phiin=phiin+sqrt(dens_initR(1))*p{1+L}'*phi0;
for j=2:L
  phiin=phiin+sqrt(dens_initH(j))*p{j}'*phi0;
  phiin=phiin+sqrt(dens_initR(j))*p{j+L}'*phi0;
end
phiin=phiin/norm(phiin);

%% Migration Matrix
MatrixR(L,L)=0;
MatrixR(1,2:4)=[1 1 1];
MatrixR(2,[3:6])=[1 1 1 1];
MatrixR(3,[5 6])=[1 1];
MatrixR(4,[5 7 8])=[1 1 1];
MatrixR(5,[6 7 8 9])=[1 1 1 1];
MatrixR(6,[8 9])=[1 1];
MatrixR(7,8)=[1];
MatrixR(8,9)=[1];

%%Hamiltonian Operators
HH{1,1}=0;
for jpop=1:L-1
  for j=jpop+1:L
    HH{jpop,j}=(p{jpop}'*p{j}+p{j}'*p{jpop}); %Migration term
  end
end

for jpop=1:L
  Hwh{jpop}=p{jpop}'*p{jpop}; %Free term for Humans
  Hwr{jpop}=p{jpop+L}'*p{jpop+L}; %Free term for Resources
  HR{jpop}= (p{jpop}'*p{jpop+L}+p{jpop+L}'*p{jpop});
     %Human-Resource interaction
end
%%%%%%%%%%%%%%%%%%%%%%%%%%%%
```

Runge Kutta numerical scheme for the Shroedinger equation (shroedingerevol.m file)

```
function [psi,T,NH,NR]=
  shroedingerevol(L,p,phiin,matrixR,HH,HR,Hwh,Hwr,tspan)
```

```
% Runge Kutta numerical scheme for the Shroedinger equation
% Input parameters are set in the MAIN.m and init.m files

%OUTPUT:
% psi:=the evolved vector psi for all time in tspan
% T:=the tspan
% NH:= human densities in the cells at each time
% NR:= densities of resources in the cells at each time

%Runge-Kutta Scheme
options = odeset('RelTol',1e-4,'AbsTol',1e-4*ones(length(phiin),1))';
[T,psi] = ode45(@(t, z) secmem(L, p, matrixR, HH, HR, Hwh, Hwr, z, t),
  tspan, phiin, options);

%Evaluation of the densities in each cell
for t=1:length(tspan)
  for j=1:L
    NH(j,t)=norm(p{j}'*p{j}*psi(t,:)')^2;
    NR(j,t)=norm(p{j+L}'*p{j+L}*psi(t,:)')^2;
  end
end

function jout=secmem(L,p,matrixR,HH,HR,Hwh,Hwr,z,t)
timescale=1000;
paramscale=80;

for j=1:L
  NH(j)=norm(p{j}'*p{j}*z)^2;
  NR(j)=norm(p{j+L}'*p{j+L}*z)^2;
  qq(j)=(NR(j)/NH(j));
  qq2(j)=(NH(j)/NR(j));
  ll(j)=sqrt(exp(-(qq(j)/0.35)^2)*(qq2(j)));
  rr(j)=sqrt(exp(-(qq2(j)/0.35)^2)*(qq(j)));
end

omega(1:2*L)=[ones(1,L).*rr ones(1,L).*ll];
lambdaexH(L,L)=0;
for jpop=1:L-1
  for j=jpop+1:L
    lambdaexH(jpop,j)=(0.00+ll(jpop)+ll(j))*matrixR(jpop,j);
  end
end

lambdaexR=ones(L,L);
mu(1:L)=ones(1,L).*(0.02+ll+rr);
H=sparse(4^(L),4^(L));

%%Inertial and interaction terms
for jpop=1:L
  H=H+omega(jpop)*Hwh{jpop}+omega(jpop+L)*Hwr{jpop}+
    mu(jpop)*(HR{jpop});
end

%%Migration terms
for jpop=1:L-1
  for j=jpop+1:L
    H=H+lambdaexH(jpop,j)*(HH{jpop,j});
  end
end

%H=hamiltoniansystem(p,L,omega,lambdaexH,lambdaexR,mu,HH,HR,Hwh,Hwr);
jout=-1i*timescale*H*z/paramscale;
%%%%%%%%%%%%%%%%%%%%%%%
```
