# Supplementary figures and images for: Large-scale effects of migration and conflict in pre-agricultural groups: Insights from a dynamic model
Source: PLoS One. 2017 Mar 8;12(3):e0172262. doi: 10.1371/journal.pone.0172262 (PMC5342208; doi:10.1371/journal.pone.0172262)

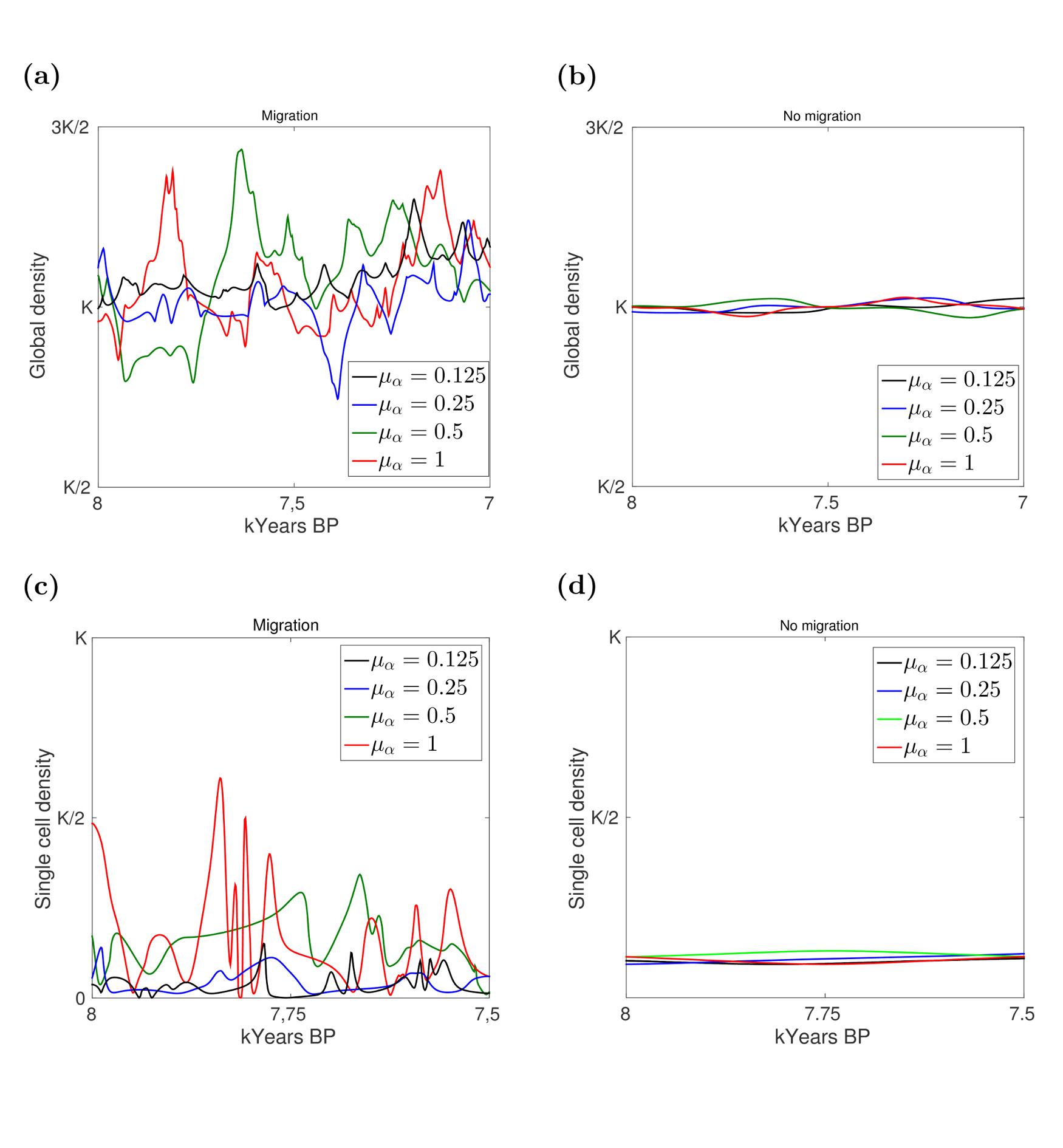

Supplement: S1 Fig — (a) Global (whole nine-cell lattice) human population density in the migration scenario. (b) Global human population density in the no-migration scenario. (c) Local (single cell) human population density in the migration scenario, with the central cell selected as example. (d) Local human population density in the no-migration scenario. (TIF) [file pone.0172262.s003.tif]

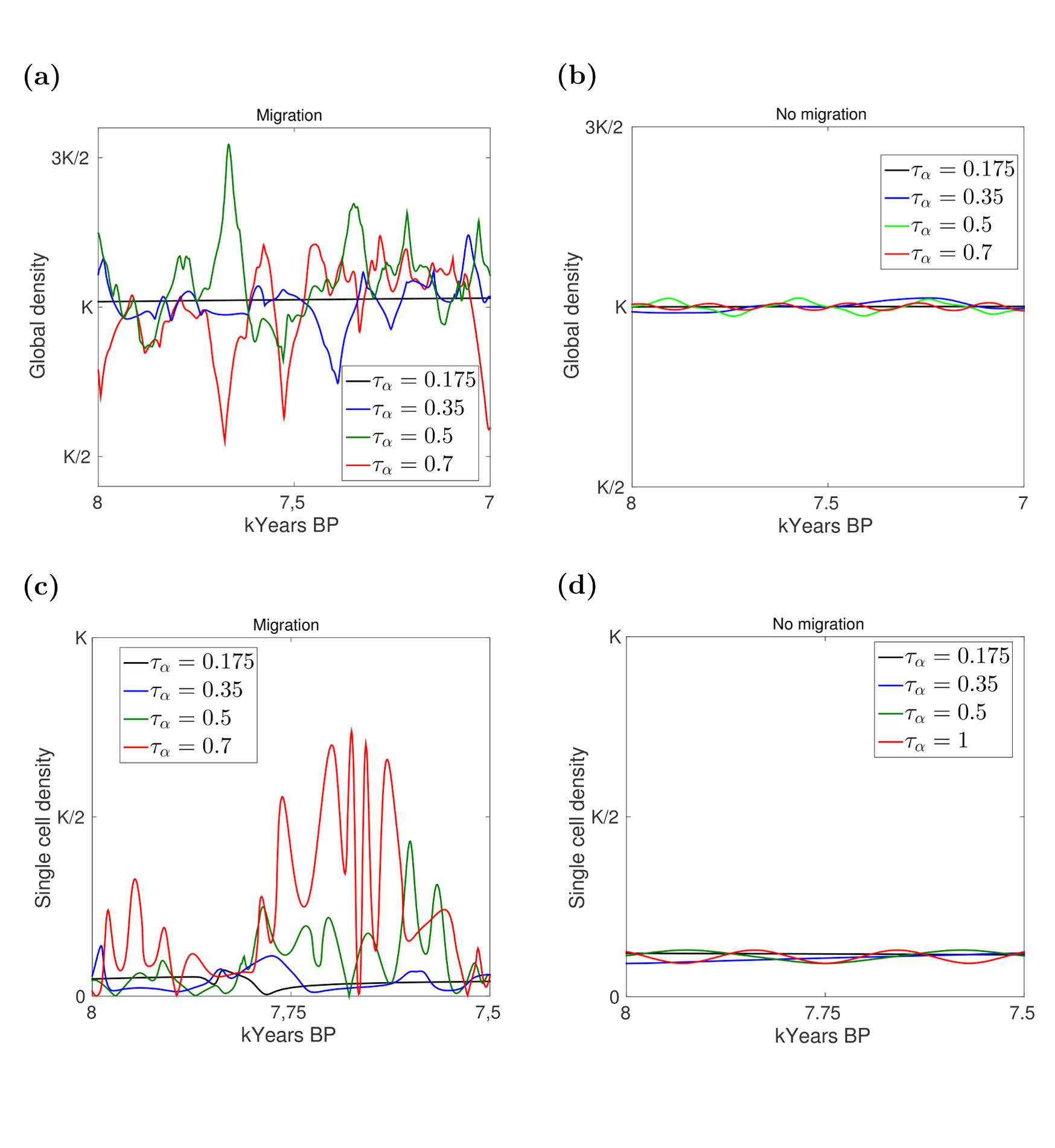

Supplement: S2 Fig — (a) Global (whole nine-cell lattice) human population density in the migration scenario. (b) Global human population density in the no-migration scenario. (c) Local (single cell) human population density in the migration scenario, with the central cell selected as example. (d) Local human population density in the no-migration scenario. (TIF) [file pone.0172262.s004.tif]

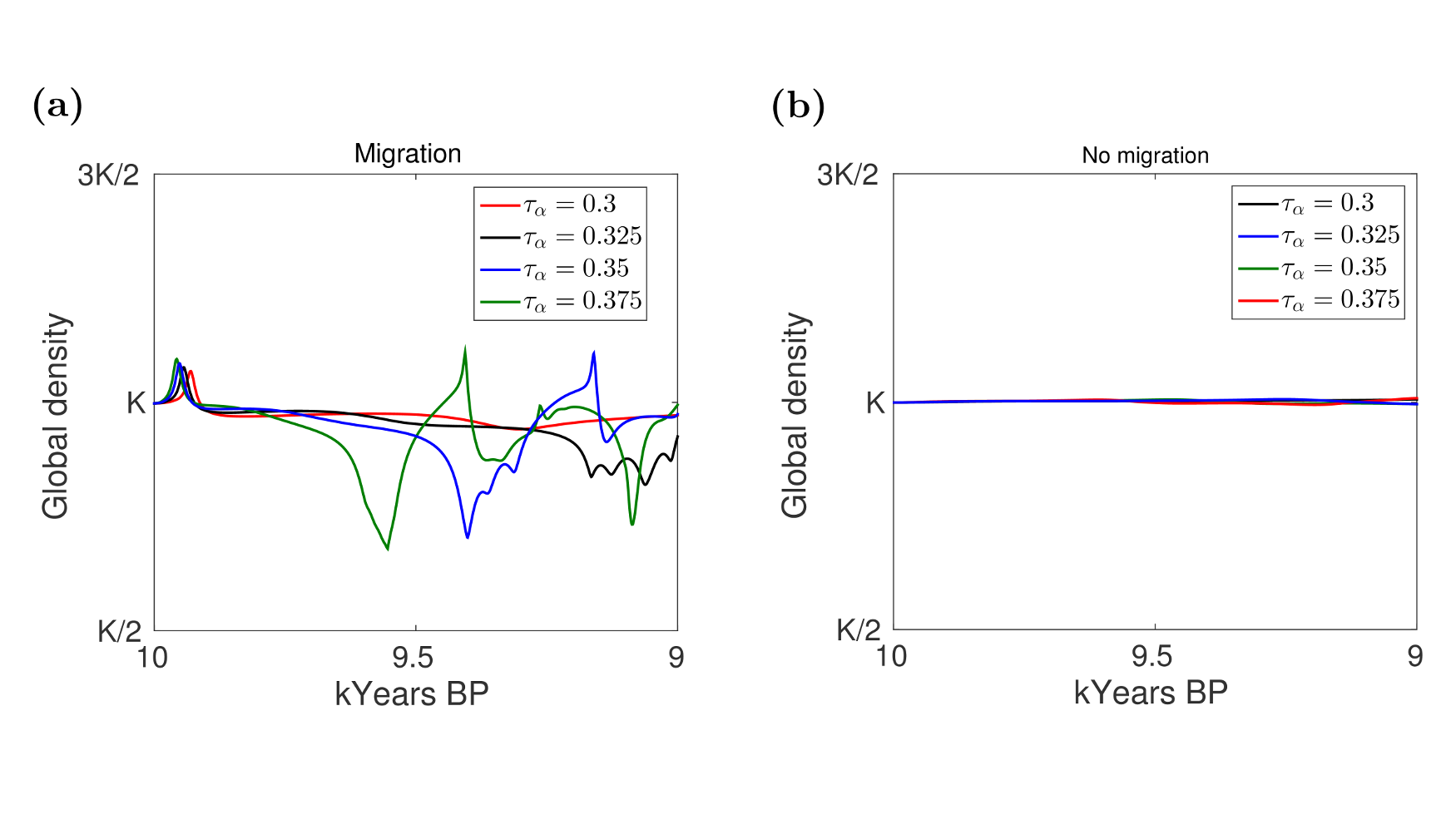

Supplement: S3 Fig — (a) Global human population density in the migration scenario. (b) Global human population density in the no-migration scenario. (TIF) [file pone.0172262.s005.tif]
